# Variant U1 snRNAs are implicated in human pluripotent stem cell maintenance and neuromuscular disease

**DOI:** 10.1093/nar/gkw711

**Published:** 2016-08-17

**Authors:** Pilar Vazquez-Arango, Jane Vowles, Cathy Browne, Elizabeth Hartfield, Hugo J. R. Fernandes, Berhan Mandefro, Dhruv Sareen, William James, Richard Wade-Martins, Sally A. Cowley, Shona Murphy, Dawn O'Reilly

**Affiliations:** 1 University of Oxford, Sir William Dunn School of Pathology, South Parks Road, Oxford, OX1 3RE, UK; 2 Oxford Parkinson's Disease Centre, University of Oxford, Oxford, UK; 3 Oxford Parkinson's Disease Centre, Department of Physiology, Anatomy and Genetics, University of Oxford, Oxford, UK; 4 Cedars-Sinai Medical Center, Board of Governors-Regenerative Medicine Institute and Department of Biomedical Sciences, 8700 Beverly Blvd, AHSP A8418, Los Angeles, CA 90048, USA; 5 iPSC Core, The David and Janet Polak Foundation Stem Cell Core Laboratory, Cedars-Sinai Medical Center, Los Angeles, CA 90048, USA

## Abstract

The U1 small nuclear (sn)RNA (U1) is a multifunctional ncRNA, known for its pivotal role in pre-mRNA splicing and regulation of RNA 3′ end processing events. We recently demonstrated that a new class of human U1-like snRNAs, the variant (v)U1 snRNAs (vU1s), also participate in pre-mRNA processing events. In this study, we show that several human vU1 genes are specifically upregulated in stem cells and participate in the regulation of cell fate decisions. Significantly, ectopic expression of vU1 genes in human skin fibroblasts leads to increases in levels of key pluripotent stem cell mRNA markers, including NANOG and SOX2. These results reveal an important role for vU1s in the control of key regulatory networks orchestrating the transitions between stem cell maintenance and differentiation. Moreover, vU1 expression varies inversely with U1 expression during differentiation and cell re-programming and this pattern of expression is specifically de-regulated in iPSC-derived motor neurons from Spinal Muscular Atrophy (SMA) type 1 patient's. Accordingly, we suggest that an imbalance in the vU1/U1 ratio, rather than an overall reduction in Uridyl-rich (U)-snRNAs, may contribute to the specific neuromuscular disease phenotype associated with SMA.

## INTRODUCTION

Precise control of expression of protein-coding genes, which is fundamental to an organism's fitness and survival, is achieved through intricate co-ordination of transcription, RNA processing and translation. Since the onset of transcriptomics, it has become increasingly evident that non-coding RNAs are key regulators of these processes (1). The pol II-transcribed Uridyl-rich small nuclear (Usn)RNA, U1, in the form of a ribonucleoprotein (RNP) complex, plays a pivotal role in regulating RNA isoform production via intimate interactions with the nascent RNA and two major RNA processing machineries, the Spliceosome and Polyadenylation Complex (2–5). The 5′ end of U1 base-pairs with complementary sequences throughout the pre-mRNA to recruit the Spliceosome to exon/intron junctions and to inhibit cleavage and polyadenylation at internal cryptic poly A (pA) sites (6–8). Thus, depending on where U1 binds, some exons can be skipped, introns included and/or internal cryptic pA sites selected to facilitate the production of a range of different proteins from individual genes. Consequently, control of U1 activity is imperative to ensure that the correct protein is made in the appropriate cell throughout development. The stoichiometry and tissue-specificity of trans-acting factors, including splicing regulators, play major roles in regulating U1 snRNP recruitment to target sites in different human cell types (9–11).

In addition to U1 genes, variant U1 snRNA genes (vU1) have been described in several non-human species, including mouse (12,13), frog (14), fly (15), moth (16) and sea urchin (17,18). Sequence analysis of these orthologues suggest they have undergone concerted evolution, i.e. the multicopy U1/vU1 gene families are more similar within a species than between species. Expression analysis indicates that vU1s are most highly expressed during the early stages of development, reaching levels close to 40% of the total U1 in some cases (12,19). As development progresses, these variants are down-regulated and the major U1 orthologues gradually dominate expression (20). This developmental switching pattern supports an important function for vU1s in regulating early cell fate decisions (21–24). However, analysis of their specific role in controlling stem cell identity has been hampered due to their high level of sequence conservation, making target-gene identification and elucidation of their mechanism(s) of action difficult.

We recently characterized a family of functional pol II-transcribed vU1 genes in human cells and demonstrated that one vU1 at least (vU1.8), participates in mRNA processing events of a select number of target genes (25). Since many vU1s contain base changes within regions known to bind U1-specific proteins and/or pre-mRNA donor splice sites, they likely play important roles in contributing to the unique alternative splicing/polyadenylation patterns associated with stem cell transcriptomes (26–28). Our findings prompted us to analyze expression patterns of human vU1s in different cell types to determine whether they have a specific role in regulating stem cell identity or a more general role in other tissues/cell lines. In this report, we demonstrate that vU1s are not only enriched in human pluripotent stem cells but, significantly, their ectopic expression in fully differentiated cells stimulates expression of the pluripotency marker genes, including NANOG and SOX2, indicating that these snRNAs can affect basic cell fate decisions. Furthermore, U1 and vU1 profiles display reciprocal patterns of regulation during cell reprogramming and differentiation of human embryonic stem cells (ESCs) with U1 levels increasing and vU1 levels decreasing during differentiation. These findings suggest that a fine balance exists between U1 and vU1 levels in human cells and that disruption of this balance could cause disease. In support of this, U1/vU1 ratios are notably altered in induced pluripotent stem cell (iPSC)-derived motor neuron cultures (MNs) from patients suffering with Spinal Muscular Atrophy (SMA) disease compared to healthy control subjects or patients suffering from other neurological disorders, including Parkinson's disease, for example. These findings lead us to speculate that the perturbations in the ratio of U1 to vU1 levels in different cell types, rather than reductions in overall levels of U-snRNAs, may underlie the pathophysiology of motor neuron disease.

## MATERIALS AND METHODS

### Plasmid construction

The U1 promoter and U1/vU1 (vU1.2, vU1.3, vU1.8, vU1.13 and vU1.20) coding sequences were polymerase chain reaction (PCR) amplified from genomic U1/vU1 constructs, previously generated in the laboratory (25). The U1 promoter fragment, U1 3′ end annealed oligonucleotides (U1 3′end F/R primers) and the U1/vU1 coding fragments were ligated into a pGEM4 plasmid. See Supplementary Table S1 for primer sequences.

### Real-time quantitative (q)PCR

Total RNA was isolated from primary human skin fibroblasts, human ESCs (HUES-1, -2, -4), Embryoid Bodies (EBs), ESC-derived monocytes, human iPSCs, iPSC-derived motor and -dopaminergic neurons from SMA and Parkinson'disease patients, respectively, using either Trizol reagent (Invitrogen) or Qiagen's (mi)RNA purification kits according to the manufacturers’ instructions. Total RNA extracted from human tissues, including fetal and adult brain, lung, spleen (fetal only), kidney, heart and placenta (adult only), was obtained from Agilent technologies. cDNA was generated from these samples using Superscript III (Invitrogen), according to manufacturers’ instructions. Real-time qPCR was performed using a QuantiTect SYBER Green mastermix (Qiagen). All oligonucleotides used in qPCR reactions are outlined in Supplementary Table S2.

### Cell culture and transfection

Human ESCs (HUES-1, -2 and -4) (passages 16–38) were obtained from the HUES Facility, University of Harvard (29,30). They were cultured in mTeSR1 medium (Stem Cell Technologies) on human ESC-qualified Matrigel (Becton Dickinson) (31).

Human skin fibroblasts from healthy controls (GM02183, GM03814, GM03815, GM05400, ND30625, NHDF (32), OX1 (33), SMA type 1 patients (GM09677, GM00232, GM03813, CS83SMA) and Parkinson's patient (JR036, Sandor *et al.*, submitted) were derived from normal human dermal fibroblasts purchased from Coriell Cell Repository (GM05400, GM02183, GM10684, GM03814, GM03815, GM09677, GM00232), Cedars-Sinai (CS83SMA), Lonza (NHDF) and from skin biopsies (4 mm diameter) from healthy control (OX1) and Parkinson (JR036) participants after signed informed consent. Human skin fibroblasts from SMA type 1 patient (GM10684) were derived from a EBV-transformed lymphoblastoid cell line. JR036-1 and JR036-2 lines were generated from a Parkinson's patient (JR036) carrying the LRRK2 G2019S mutation. All fibroblasts were cultured in advanced Modified Eagle's Media (Invitrogen) supplemented with 10% Fetal Calf Serum, 1% non-essential amino acids, 110 mg/l Sodium Pyruvate and 4 mM L-Glutamine.

Human NHDF fibroblasts were transfected with the indicated dose(s) of plasmid for 24 h using Lipofectamine® 2000 reagent (Thermo Fisher Scientific) according to the manufacturers’ instructions. All samples were normalized to 1 μg with pGEM4 control vector.

### Generation and characterization of human iPSC lines

Non-integrating iPSC lines were generated from fibroblasts, taken from SMA and matched healthy control subjects, at Cedars-Sinai using the episomal plasmid method of reprogramming as described previously (34). Briefly, iPSC lines were reprogrammed from dermal fibroblasts into virus-free iPSC lines with the Lonza Nucleofector Kit using an episomal plasmid (Addgene) expressing 6 factors: *OCT4, SOX2, KLF4, L-MYC, LIN28* and *p53* shRNA (pCXLE-hOCT3/4-shp53-F, pCXLE-hUL and pCXLE-hSK). Dermal fibroblasts (1 × 10^6^ cells per nucleofection) were harvested, centrifuged at 1500 rpm for 5 min, re-suspended carefully in Nucleofector® Solution and the U-023 program was applied. These nucleofected cells were plated on feeder-independent BD Matrigel™ growth factor-reduced Matrix (Corning/BD Biosciences, #354230). Individual iPSC colonies with ESC/iPSC-like morphology appeared between day 25–32 and those with best morphology were mechanically isolated, transferred onto 12-well plates with fresh Matrigel™ Matrix, and maintained in mTeSR®1 medium. The iPSC clones from SMA and control subject lines were further expanded and scaled up for further analysis.

All other iPSC lines (Parkinson's disease and additional matched healthy control patients) were derived from skin biopsies or monocytes and reprogrammed in the same laboratory as described previously (33,35). iPSC lines were grown in mTeSR1 on Matrigel (Corning)-coated tissue culture dishes, passaged using 0.5 mM EDTA or using TrypLE, and plated with the Rho-kinase inhibitor Y-27632 (10 μmol/l). Cells were frozen in SNP-QCed batches of at least 30 vials (within a narrow window of passages, typically 15–30), from which cells would be thawed for each experiment, to ensure consistency across experiments.

### Generation of human ESC-derived monocytes

Human ESCs were first differentiated to EBs by dissociation with TrypLE Express (Invitrogen). A total of 300 EBs were generated from ∼4 × 10^6^ cells, aggregated in mTeSR1 medium with 10 mM ROCK inhibitor Y-27632 (Calbiochem) by spinning in an AggreWell (Stem Cell Technologies) according to the manufacturer's manual. The EBs were analyzed at day 4 post-aggregation. For subsequent myeloid differentiation, EBs were cultured in medium consisting of X-VIVO^TM^15 (Lonza), supplemented with 100 ng/ml Macrophage colony-stimulating factor (M-CSF) (Invitrogen), 25 ng/ml Interleukin 3 (IL-3) (R&D), 62 mM Glutamax (Invitrogen), 100 U/ml Penicillin and 100 μg/ml Streptomycin (Invitrogen) and 0.055 mM β-Mercaptoethanol (Invitrogen). Once ESC-derived monocytes were visible in the supernatant of the cultures (from 2–3 weeks onward), the non-adherent monocytes were harvested weekly, as has been described previously (36). Monocytes released from the ‘factories’ were used directly.

### Generation of human iPSC-derived motor neuron cultures (MNs)

The iPSCs were grown to near confluence under normal maintenance conditions before the start of the differentiation as per protocols described previously (34,37–39). Briefly, iPSCs were gently lifted by Accutase treatment for 5 min at 37°C. A total of 1.5–2.5 × 10^4^ cells were subsequently placed in each well of a 384 well plate in defined neural differentiation medium with dual-SMAD inhibition (0.2 μM LDN193189 and 10 μM SB431542). After 2 days, neural aggregates were transferred to low adherence flasks. After 6 days, neural aggregates were plated onto Laminin-coated 6-well plates to induce rosette formation. From day 12–18, the media was supplemented with 0.1 µM Retinoic Acid and 1 μM Purmorphamine along with 20 ng/ml Brain-Derived Neurotrophic Factor (BDNF), 200 ng/ml Ascorbic Acid, 20 ng/ml Glial-derived neurotrophic factor (GDNF) and 1 mM dbcAMP and neural rosettes were selected using rosette selection media (Stemcell Tech, 05832). The purified rosettes were subsequently supplemented with 100 ng of Epidermal Growth Factor (EGF) and Fibroblast Growth Factor (FGF). These neural aggregates were expanded over a 2–7 week period, disassociated with Accutase and then plated onto Laminin-coated plates. These MN precursors were terminally differentiated over 21 days period prior to harvest using the MN maturation media consisting of Neurobasal supplemented with 1% N2, Ascorbic Acid (200 ng/ml), Dibutyryl Cyclic Adenosine Monophosphate (1 μM), BDNF (10 ng/ml) and GDNF (10 ng/ml).

### Generation of human iPSC-derived dopaminergic neuron cultures (DNs)

Prior to differentiation, iPSC lines were adapted to feeder-free conditions using Matrigel (BD) and EBs were formed. After 4 days, neural induction was initiated as previously described (32,40). Briefly, EBs were plated onto Geltrex-coated plates in Neural Induction medium 1 (DMEM/F12 supplemented with L-Glutamine (2 mM), N2 supplement, bovine serum albumin (BSA) (1 mg/ml), Y27632 (10 μM; Tocris), SB431542 (10 μM, Tocris), Noggin (200 ng/ml) and antibiotic/antimycotic (1% v/v)). After 4 days, medium was changed to Neural Induction medium 2 (as NI1, without SB431542 and Noggin and with addition of SHH C24II (200 ng/ml; SHH C24II; R&D Systems). After 6 days, medium was supplemented with FGF8a (100 ng/ml; R&D Systems), Heparin (5 μg/ml; Sigma), BDNF (20 ng/ml) and Ascorbic Acid (200 μM; Sigma)) and incubated for 7 days, until the appearance of dense neural rosette structures. Neural progenitor cells were manually selected and re-plated onto Poly-D-Lysine/Laminin-coated plates in final differentiation medium (DMEM/F12 supplemented with L-Glutamine (2 mM), N2 supplement, BDNF (20 μg/ml), GDNF (20 μg/ml), N6, 2′ -O-dibutyryladenosine 3′,5′ -cyclic monophosphate sodium salt (dcAMP, 0.5 mM; Sigma), Laminin (1 μg/ml) and antibiotic/antimycotic (1% (v/v)). Neurons were matured for 2 weeks in this medium before experimental procedures were carried out.

### Characterization of iPSC lines and iPSC-derived neuron cultures

To assess for genome integrity, iPSCs derived from SMA and healthy control patients were incubated in Colcemid (100 ng/ml; Life Technologies) for 30 min at 37°C, dissociated using TrypLE for 10 min and washed in phosphate buffered saline (PBS). Following incubation at 37°C in 5 ml hypotonic solution (1 g KCl, 1 g Na Citrate in 400 ml water) for 30 min, cells were centrifuged for 2.5 min at 1500 rpm and resuspended in fixative (Methanol: Acetic Acid, 3:1) at RT for 5 min. This was repeated twice, and cells were resuspended in 500 μl of fixative solution and submitted to the Cedars-Sinai Clinical Cytogenetics Core for G-Band karyotyping. In addition, to assess for expression of pluripotency and neuronal markers on iPSC-derived motor neuronal cultures (MNs), immunohistochemistry analysis was performed as follows; patient-derived iPSC lines and MNs were plated on glass coverslips or optical-bottom 96-well plates (Thermo, Catalog # 165305) and subsequently fixed in 4% Paraformaldehyde. All cells were blocked in 5% normal donkey serum with 0.1% Triton X-100 and incubated with primary antibodies (Supplementary Table S4) either for 1 h at RT or overnight at 4°C. Cells were then rinsed and incubated in species-specific AF488, AF594 or AF647-conjugated secondary antibodies followed by Hoechst 33258 (0.5 μg/ml; Sigma) to counterstain nuclei. Cells were imaged using Molecular Devices Image Express Micro high-content imaging system or using Leica microscopes.

iPSC and iPSC neuronal cultures derived from healthy control and Parkinson's disease patients were characterized as previously described (32). Briefly, iPSCs were analyzed by fluorescence-activated cell sorting (FACS) analysis to assess expression of pluripotency markers and quantitative reverse transcription-PCR (qRT-PCR) analysis to assess silencing of retroviral transgene sequences. In addition, genome integrity and karyotype analysis was assessed by an Illumia Human CytoSNP-12v2.1 beadchip (∼300 000 markers), which was analyzed using KaryoStudio software (Illumina). iPSC derived dopaminergic neuronal cultures were assessed by expression of neuronal marker Tuj1 (β-tubulin III), the dopaminergic marker TH (Tyrosine Hydroxlase) and AADC (amino acid decarboxylase). To further confirm the dopaminergic function of derived neurons, Dopamine content was also analyzed by High Performance Liquid Chromatography (HPLC) as previously described (32).

### Characterization of human iPSC lines, JR036-2 and OX1-40

Previously uncharacterized iPSC lines (JR036-2 and OX1-40) were characterized as described previously (35,40). Briefly, genome integrity was assessed by Illumina Human CytoSNP-12v2.1 beadchip array (∼300 000 markers) and analyzed using Karyostudio to generate karyograms and Genomestudio software (Illumina) to track samples to confirm parentage. Pluripotent protein expression used antibodies to TRA-1-60 (B119983, IgM-488, Biolegend) for immunocytochemistry or FACs, and Nanog (2985S, IgG-647, Cell Signaling) for FACs, with appropriate isotype control, at the same concentration, from the same supplier. Cells were fixed for 10 min in 2% paraformaldehyde in PBS (Alfa Aesar), permeabilized in 100% methanol at −20°C for at least 30 min before staining and acquisition on a FACS Calibur (Becton Dickinson), analyzed with FlowJo software. qRT-PCR was used to assess silencing of Retrovirus-delivered reprogramming genes, using fibroblasts as negative controls and fibroblasts infected 5 days previously as positive controls. Lines reprogrammed with non-integrating Sendai virus SeVdpmir302L automatically clear residual virus from the cytoplasm within ∼6 passages because the virus contains a binding site for mir302, which is expressed in pluripotent stem cells. Illumina HT12v4 Transcriptome array data sets, generated from RNA extracted from iPSC lines, were uploaded to Pluritest.org to generate Pluritest plots, to assess conformity of the iPSc lines to a pluripotent phenotype.

### Knockdown experiments

2′-O-methyl (2′OMe) RNA/DNA oligonucleotides were purchased from Integrated DNA Technologies (IDT). Five nucleotides at the 5′ and 3′ ends were substituted with 2′-O-Methyl ribonucleotides. All bases were phosphorothioate converted. The control oligonucleotide is a scrambled sequence of 21 bases, the U1-, vU1.8- and vU1.20-specific oligonucleotides are antisense to U1 at position 1 to 25, vU1.8 at position 11 to 33 and, vU1.20 at position 9 to 31, respectively, where 1 is the first base of the U1/vU1 snRNA sequence. See Supplementary Table S3 for oligonucleotide sequences. Knockdown experiments were performed in HeLa cells with Lipofectamine 2000 reagent according to manufacturers’ instructions. Typically, 1 × 10^7^ cells were transfected with 600 pmoles of the 2′-OMe oligonucleotide and cells harvested 18 h later.

### Flow cytometry

Cells were washed and stained in FACS buffer consisting of PBS, human IgG (10 μg/ml, Sigma), 1% Fetal Calf Serum (Hyclone) and 0.01% Sodium Azide as previously described (36). For intracellular staining, cells were fixed in 2% Formaldehyde, permeabilized with 0.2% Saponin and stained for 45 to 60 min. Cells were washed three times before acquisition and primary antibodies were compared with an isotype-matched control. Antibodies used included, IgG and NANOG (Alexa Fluor647 conjugated)(Cell signaling). Data were analyzed using FlowJo software and presented as histograms with antibody staining in black relative to isotype-matched control in gray. The percentage of cells expressing markers was determined by subtracting the isotype background from the antibody staining percentage.

### Western blot analysis

Western blot analysis was carried out essentially as described in (32).

### High performance liquid chromatography (HPLC)

Dopamine content was analyzed using HPLC with electrochemical detection essentially as described previously (32).

### Ethics statement

The human ESC lines HUES-1, 2 and -4 were obtained from the HUES Facility, University of Harvard (39). Ethical approval for work on all hES cell lines was reviewed and approved by the UK Stem Cell Bank Steering Committee.

NDHF fibroblasts were obtained from Lonza who provided the following ethics statement: these cells were isolated from donated human tissue after obtaining permission for their use in research applications by informed consent or legal authorization. The human iPSC lines derived from Lonza fibroblasts were generated as control lines for part of a larger-scale project – Healthy and Parkinson's disease patient participants were recruited to this study having given signed informed consent that included mutation screening and derivation of iPSC lines (Ethics Committee: National Health Service, Health Research Authority, NRES Committee South Central, Berkshire, UK, who specifically approved this part of the study (REC 10/H0505/71). All patients included in the study fulfilled UK Brain Bank diagnostic criteria for clinically probable PD at presentation (41).

Healthy and SMA patient dermal fibroblast cell lines and EBV-transformed lymphoblastoid cell lines (LCL) were obtained from the Coriell Institute for Medical Research. The Coriell Cell Repository maintains the consent and privacy of the donor fibroblasts and LCLs. All the cell lines and protocols in the present study were carried out in accordance with the guidelines approved by stem cell research oversight committee (SCRO) and institutional review board (IRB) at the Cedars-Sinai Medical Center under the auspice IRB-SCRO Protocols Pro00032834 (iPSC Core Repository and Stem Cell Program), Pro00024839 (Using iPS cells to develop novel tools for the treatment of Spinal Muscular Atrophy) and Pro00036896 (Sareen Stem Cell Program). All the cell lines and protocols in the present study were carried out in accordance with the guidelines approved by institutional review boards at the Cedars-Sinai Medical Center, Washington University at St. Louis, USA.

## RESULTS

### Human vU1 genes are specifically upregulated in undifferentiated stem cells

**Figure 1.**
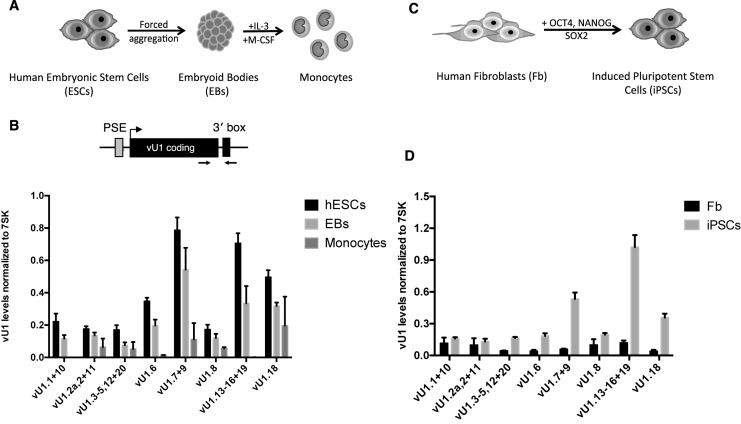
vU1 gene expression is regulated throughout differentiation and cell re-programming. (**A**) Schematic of the protocol used for the gradual differentiation of human ESCs (HUES2) into monocytes. (**B**) Expression profiling of nascent vU1 levels in HUES2, Embryoid bodies (EB) and HUES2-derived monocytes, by quantitative reverse-transcription (qRT)-PCR analysis. vU1 levels were estimated using a gDNA as standard and normalized to 7SK levels across the different cell types. The position of the primers is indicated in the schematic. Regulatory elements, known to be required for U1/vU1 expression (proximal sequence element (PSE) and 3′ end processing (3′box)) are noted on the schematic. vU1 genes that show a greater than 95% sequence identity are grouped. Error bars represent standard error of the mean (SEM) of 3 independent differentiation experiments (two-way ANOVA analysis, * = *P* < 0.1, ** = *P* < 0.05, *** = *P* < 0.001, **** = *P* < 0.0001 and one-way ANOVA analysis (vU1.8 and vU1.3-5,12+20 genes only); * = *P* < 0.05. (**C**) Schematic of the protocol used for the de-differentiation of human skin fibroblasts into pluripotent stem cells. (**D**) Expression profiling of nascent vU1 levels in human skin fibroblasts (Fb) and fibroblast-derived induced pluripotent stem cells (iPSCs), by qRT-PCR analysis. Primers used are illustrated in the schematic as in (B). Error bars represent SEM of 3 independent re-programming experiments (two-way ANOVA analysis (vU1.7+9, vU1.13–16+19 and vU1.18); **** = *P* < 0.0001 and one-way ANOVA analysis; * = *P* < 0.05).

We quantified the expression of nascent vU1 levels in human ESCs and following directed differentiation into monocytes to determine the relationship between expression of these snRNA genes and stem cell identity/differentiation. In agreement with our previous study, expression analysis of representative members of each group of vU1 genes indicates that different vU1s are expressed at varying levels in the undifferentiated human ESCs (25) (Figure 1A). For example, some vU1 genes, including vU1.7+9, vU1.13–16+18 and vU1.18, express snRNAs at levels ranging from 2- to 4-fold greater than levels produced from vU1.1+10, vU1.2a, 2+11, vU1.6, vU1.3–5,12+19 and vU1.8 genes. This pattern of expression is not unique to this ESC line (HUES2), as similar levels of vU1s are expressed in additional human stem cell lines, including HUES1 and HUES4 (29) (Supplementary Figure S1). Note, the efficiency of PCR amplification for each primer pair was controlled using a genomic DNA as standard.

We next differentiated the HUES2 cells into EBs and thereafter into non-adherent monocytes, using a directed differentiation protocol, which was developed in-house for the routine production of authentic macrophages from human pluripotent stem cells (36). EB cultures, treated with M-CSF and IL-3, start differentiating into monocytes following a lag period of ∼12 days and continue to produce monocytes over a period of months. Harvested monoctyes routinely undergo validation in the laboratory by FACS analysis for expression of monocytic-specific surface markers (33). As an additional validation, we quantitated changes in expression profiles of a subset of myeloid cell surface receptors, including CD14 and CD68 and markers specifically expressed in the human ESC cultures, for example OCT4 (42–44). As illustrated in Supplementary Figure S2, human ESCs and EBs express high levels of OCT4 mRNA with levels dramatically reduced following differentiation. In contrast, CD14 and CD68 levels are low in human ESCs and EBs but increase markedly with differentiation toward the macrophage lineage, as expected. We next assessed whether vU1 gene expression also changes during differentiation. As illustrated in Figure 1B, there is a marked reduction in the levels of nascent vU1s following directed differentiation of human ESCs into monocytes. A decrease in nascent vU1 levels is already apparent at the first differentiation stage (EB formation) and expression from all vU1 genes continues to fall as the human ESCs progress through differentiation into monocytes.

We further investigated whether up-regulation of vU1 expression is a characteristic feature of undifferentiated cells by analyzing changes in vU1 expression in iPSCs derived from re-programming human skin fibroblasts (Figure 1C). In line with the results of analysis of human ESCs and human ESC-derived monocytes, levels of all vU1s are low in the fully differentiated human skin fibroblasts and increase markedly upon iPSC generation. The pattern of vU1 expression in iPSCs is similar to that in human ESCs (Compare Figure 1B and D). Thus, vU1 gene expression appears to change during the different stages of human ESC differentiation and cell re-programming.

### Differential regulation of U1 and vU1 expression following differentiation

**Figure 2.**
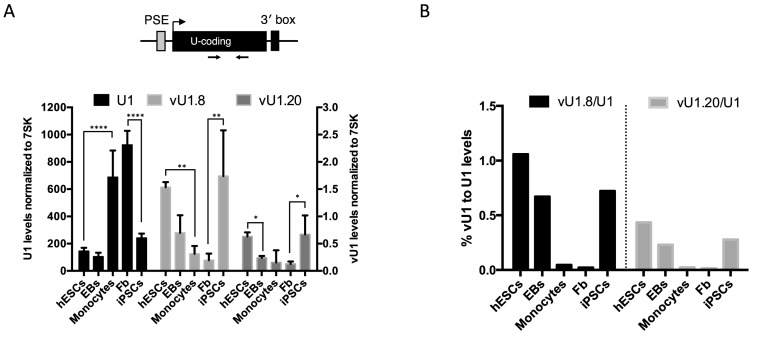
vU1 and U1 genes follow reciprocal patterns of expression during differentiation and cell re-programming. (**A**) qRT-PCR analysis of steady state U1, vU1.8 and vU1.20 levels in human ESCs, EBs, ESC-derived monocytes, human skin fibroblasts and fibroblasts-derived iPSCs. Primers used are illustrated in the schematic above the graph. U1/vU1 levels were estimated using a genomic (g)DNA as standard and normalized to 7SK levels across the different cell types. U1 levels are indicated on the Y-axis to the left of the graph and vU1 levels on the right Y-axis. Error bars represent SEM of three independent differentiation/re-programming experiments (Two-way ANOVA analysis (U1); **** = *P* < 0.0001 and one-way ANOVA (vU1.8 +vU1.20); ** = *P* < 0.01, *P = 0.05. (**B**) The ratio of vU1 to U1 levels, expressed as a percentage of U1 levels, across the different cell types.

To determine whether changes in the levels of nascent vU1s during differentiation are reflected in the levels of functionally mature vU1s, we analyzed expression of steady state levels of U1 and vU1s in the different cell types outlined above. Unlike the vU1 3′ flanking regions, which diverge significantly from U1 enabling accurate measurement of nascent levels expressed from several vU1 genes, few vU1 differ significantly in their non-coding RNA regions to allow specific amplification of their mature snRNAs (25). Consequently, two vU1s (vU1.8 and vU1.20) were chosen for further analyses as they are sufficiently divergent from each other and U1 to allow specific selection. As illustrated in Figure 2A and Supplementary Figures S3 and S4, changes in vU1 expression lead to corresponding changes in the production of steady state vU1s. Both vU1.8 and vU1.20 levels are high in human ESCs and iPSCs, and markedly lower in human skin fibroblasts and human ESC-derived monocytes, as expected. Interestingly, U1 shows an altered pattern of expression. U1 steady state levels are 4-fold higher in human monocytes and skin fibroblasts than in human ESCs, EBs and iPSCs (Figure 2A and Supplementary Figure S3). Moreover, further analysis of U1 levels across a panel of fetal and adult tissues confirms the enrichment of U1 in the differentiated cell types only (Supplementary Figure S5). Importantly, quantitating vU1 levels as a percentage of U1 levels across the different cell types indicates a significant enhancement of vU1s in stem cells specifically (vU1.8 and vU1.20 represent 1.0% and 0.4% U1 levels, respectively) compared to relative levels quantitated in the differentiated monocytes/fibroblasts (vU1.8 and vU1.20 represent 0.04%/0.02% and 0.02%/0.01% U1 levels, respectively) (Figure 2B). These levels are similar to the levels reported for the minor Spliceosome in some tissues (∼1%), re-enforcing the idea that vU1s have the potential to impact regulatory networks controlling stem cell identity. Our findings are the first to demonstrate that U1 levels are significantly reduced in stem cells, which is consistent with recent findings indicating a marked increase in the production of short polyadenylated transcripts specifically in these cell types (45,46). Altering the ratio of vU1 to U1 levels may be a crucial step for the effect of vU1s on stem cell maintenance and/or pluripotency. In line with the idea that the levels of U1 and vU1 are co-regulated, targeted knockdown of U1, using antisense oligonucleotides, results in a 2-fold increase in vU1.8 and vU1.20 levels (Supplementary Figure S6). These findings suggest that feedback mechanisms exist to maintain the correct balance of vU1/U1 levels in different cells.

### vU1s promote expression of key pluripotent stem cell marker genes

**Figure 3.**
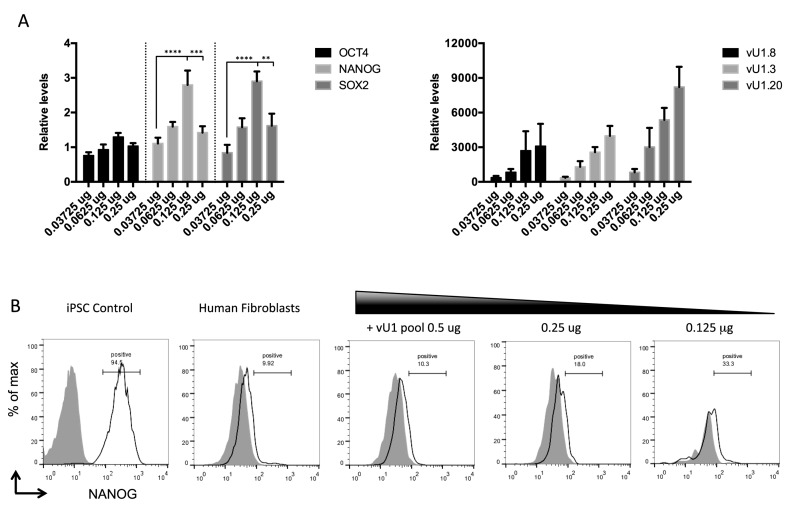
vU1s participate in early cell fate decisions. (**A**) Steady state levels of pluripotent stem cell marker mRNAs, including OCT4, NANOG and SOX2, were measured following transfection of human fibroblasts (NHDF) cells with increasing doses (0.0375, 0.0625, 0.125 and 0.25 μg) of a mixed pool of vU1-expressing plasmids (vU1.2, vU1.3, vU1.8, vU1.13 and vU1.20), by qRT-PCR analysis. Changes in pluripotent mRNA levels (**left graph**), and vU1 levels (that could be specifically amplified) (**right graph**), are expressed as fold-difference over levels quantitated in cells transfected with control vector alone, which is set to 1.0. Error bars represent SEM of three independent transfection experiments (Two-way ANOVA analysis; ** = *P* > 0.05, *** = *P* > 0.001, **** = *P* > 0.0001). (**B**) FACS analysis of NANOG expression in human fibroblasts transfected with decreasing doses of the pooled vU1 plasmids, including 0.5, 0.25 and 0.125 μg. iPSCs and pGEM4 transfected human fibroblast (NDHF-1) cells were used as positive and negative controls, respectively. Histograms represent NANOG fluorescence (black line) compared to isotype control (shaded gray). The % of NANOG positive cells is noted in each histogram.

Our previous data indicate that vU1.8 regulates mRNA processing events (25) and our current work highlights a potential function of vU1s in human ESC pluripotency and cell reprogramming. If vU1s are indeed involved in regulating basic cell fate decisions, altering their levels should affect the networks that underpin the transitions between ESC pluripotency and/or differentiation. We have therefore analyzed changes in steady state levels of the key pluripotent mRNA markers following ectopic expression of vU1s in primary human skin fibroblasts. To assess their potential role in regulating stem cell identity, a number of vU1s were chosen for further analysis, including vU1.2, vU1.3, vU1.8, vU1.13 and vU1.20, based on their sequence variability from each other and U1 (25). To allow for equal expression and proper RNA 3′ processing following transfection into human fibroblasts, the corresponding snRNA-encoding regions were PCR amplified from genomic DNA and cloned into a pGEM4 vector containing the U1 promoter and 3′ flanking regions. Results illustrated in Figure 3 demonstrate that the steady state levels of mRNAs for pluripotent stem cell markers, in particular NANOG and SOX2, rise significantly (∼3-fold) (*left graph*) following the ectopic expression of increasing doses of vU1s in the primary fibroblasts (*right graph*). The same increase in NANOG and SOX2 mRNA levels is not consistently observed when vU1s are overexpressed individually (Supplementary Figure S7).

To demonstrate that this 3-fold increase in pluripotent marker mRNA levels leads to changes in protein levels, we performed FACS analysis with antibodies targeting the NANOG protein specifically. Figure 3B confirms that a partial shift in the FACS profile was observed for human fibroblasts transfected with varying doses of a mixed population of vU1 snRNA-expressing genes, at the same doses where an increase in mRNA levels are also detected (0.125 μg and 0.25 μg). These data demonstrate that vU1s are involved in the maintenance of the pluripotent stem cell state, and no single vU1 is sufficient for this regulation. Moreover, the precise level of vU1 expressed appears to be important. Too low or too high a level has little or an inhibitory effect, respectively, on the steady state levels of mRNA for pluripotency markers (Figure 3A), which suggest that mechanism(s) are in place to ensure that the appropriate stoichiometry of vU1s is maintained in different cell types. The drop-off in levels of the pluripotency mRNA markers at the highest vU1 doses transfected, is likely a consequence of variations in the abundance of other functional U-snRNPs owning to competition for general factors, including Sm proteins, for example (25). Further analysis will be required to find the correct complement of vU1s and their stoichiometry, which could enable their use for protein-free cell re-programming.

### Deregulation of vU1 expression is associated with spinal muscular atrophy

Expression of mutant U-snRNAs or alterations in their levels and/or repertoires in different human cell types can lead to disease (47–50). The best-known example of a U-snRNA associated disease is SMA. SMA is caused by the loss of the ubiquitously expressed survival motor neuron 1 gene (*SMN1*) that results in changes in cellular U-snRNA levels due to its role in U-snRNA biosynthesis (51). Motor neurons appear particularly sensitive and the molecular basis for this pathology is still unclear (52). Numerous reports have proposed that reductions in U-snRNA levels and/or U-snRNP assembly, in particular U1 and/or U11, are key to SMA pathology, owning largely to their role in mRNA processing (53–56). However, reductions in U1 levels will lead to changes in vU1/U1 ratios. Since many vU1s have the potential to recognize non-canonical splice junctions, an imbalance between U1 and vU1s, specifically in motor neurons, could result in the synthesis of novel RNA isoforms that contribute to the disease. In agreement with this idea, RNA-seq data from spinal cords extracted from the SMA mouse model indicate a high proportion of aberrant splicing defects, including RNA isoforms containing non-canonical splice site junctions and novel RNA isoforms that do not conform to normal splicing algorithms (53).

**Figure 4.**
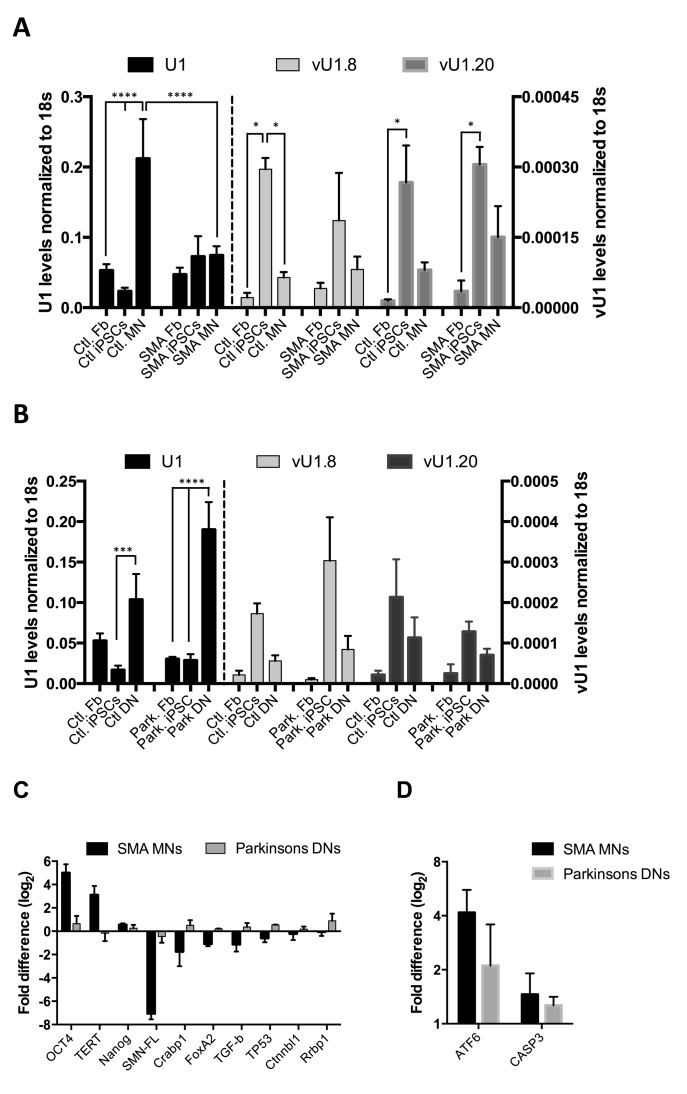
Human vU1s are implicated in SMA. (**A**) qRT-PCR analysis of U1, vU1.8 and vU1.20 levels in healthy control (Ctl) and SMA patient's skin Fb, iPSCs and MNs. U1 levels are indicated on the Y-axis to the left of the graph and vU1 levels on the right Y-axis. Error bars represent SEM of 2 independent repeats (n = 4) (Two-way ANOVA analysis; **** = *P* > 0.0001 and one-way ANOVA analysis (vU1.8 and vU1.20 genes only); * = *P* < 0.05). (**B**) qRT-PCR analysis of U1, vU1.8 and vU1.20 levels in Ctl and Parkinson's patient skin Fb. iPSCs and DNs. U1 levels are indicated on the Y-axis to the left of the graph and vU1 levels on the right Y-axis. Error bars represent SEM of 2 independent repeats (n = 4) (Two-way ANOVA analysis; *** = *P* > 0.001, **** = *P* > 0.0001). (**C**) The levels of OCT4, TERT, NANOG, SMN (SMN-FL), CRABP1, FoxA2, TGF-β, CTNNBL1 and RRBP1 transcripts were measured in total RNAs extracted from SMA MNs and Parkinson's disease patient DNs. The magnitude of change (Fold difference (log2)) in SMA MNs and Parkinson disease DNs, relative to healthy controls MNs and DNs, respectively, as determined by qRT-PCR analysis. Levels were normalized to 18s rRNA across the different cell types. Error bars represent standard error of the mean (SEM) of 2 independent repeats (n = 4). (**D**) The magnitude of change of ATF6 and CASP3 transcripts (Fold difference (log2)) in SMA MNs and Parkinson disease DNs, relative to corresponding healthy controls MNs and DNs, respectively, as determined by qRT-PCR analysis. Levels were normalized to 18s rRNA across the different cell types. Error bars represent SEM of 2 independent repeats (n = 4).

To establish whether vU1 levels are also altered in neurons from SMA patients, we profiled their patterns of expression in different cells from healthy control and SMA patients. Non-integrating iPSCs were generated from human skin fibroblasts isolated from type 1 SMA patients and healthy controls and differentiated down the motor neuronal lineage as previously described (34,37–39,57). Positive immunostaining confirms the presence of nuclear and surface pluripotency antigens, along with normal G-band karyotype, in the iPSC lines generated from SMA patient fibroblasts (Supplementary Figure S8A–D). In addition, the iPSC-derived MNs contain pan-neuron marker β_3_-tubulin (>60%) and are mostly SMI32 positive motor neurons (Supplementary Figure S8E–G). As expected, the vU1.8 and vU1.20 expression profiles vary inversely with U1 expression during re-programming of the healthy control fibroblasts into MNs, as assessed by qRT-PCR analysis (Figure 4A). Moreover, U1 levels are largely unaffected in primary fibroblasts isolated from both healthy control and SMA patients, but show approximately a 3-fold reduction in iPSC-derived MNs from SMA patients compared to healthy controls. In contrast, vU1 levels remain high, or are marginally increased, in MNs from SMA patients compared to patient fibroblasts and healthy control MNs. This significant reduction in U1 levels results in a shift in the vU1 to U1 balance in the SMA MNs in favor of the vU1s. Interestingly, U1 levels do not appear to be significantly reduced in SMA iPSCs as much as they are following reprogramming of control patient's fibroblasts that could suggest that the defects may manifest very early in development.

To determine whether this U1/vU1 imbalance is observed when iPSC cultures are driven down a different neuronal lineage or is a global characteristic of a disease state, we tested whether similar changes were observed in a Parkinson's disease model. This disease is the second most common neurodegenerative disorder characterized by the preferential degeneration of dopamine neurons in the substantia nigra pars compacta (SNpc) (58). iPSCs were derived from healthy control and Parkinson's disease patients (Supplementary Figure S9) and differentiated down the dopaminergic neuronal lineage as previously described (32). Positive immunostaining confirms the presence of Tyrosine hydrogenase (TH) and β_3_-tubulin (Tuj1) markers on the surface of representative iPSC-derived DNs (Supplementary Figure S10A). In addition, Western blot analysis confirms that the iPSC-derived DNs from healthy control and Parkinson's patient fibroblasts express key dopaminergic neuronal markers, including amino acid decarboxylase (AADC) and TH, and respond to L-DOPA (Supplementary Figure S10B and C).

Data illustrated in Figure 4B demonstrate that both U1 and vU1 have similar patterns of expression in fibroblasts, iPSCs and iPSC-derived DNs, isolated from both healthy controls and Parkinson's patients, as observed in iPSC-derived MNs from healthy control patients (Figure 4A). Although there is a significant increase in U1 levels in Parkinson's patient DNs compared to the levels in fibroblasts, there is no overall change in vU1/U1 ratios as observed in MNs from SMA patients (Supplementary Figure S11). This finding may be expected as mis-regulation of U-snRNAs levels is not associated with the pathophysiology of Parkinson's neurodegenerative disease (59). Interestingly, the switch in the ratio of U1 to vU1 levels, specifically in the SMA-derived MNs, is similar to that observed following de-differentiation of human skin fibroblasts (Figure 2A and B). Therefore, it would seem that the MNs from SMA patients adopt a vU1/U1 signature that is reminiscent of pluripotent stem cells, suggesting that defects in neuronal development are a primary underlying cause of SMA disease.

To investigate whether spinal motor neuron development is abnormal in SMA patients specifically, we measured changes in the levels of selected transcripts between the different disease models by qRT-PCR analysis. We selected the *SMN1* gene as a positive control for SMA MNs, four transcripts known to be associated with pluripotency (OCT4, Nanog, *Telomerase reverse transcriptase* (TERT) (60) and Tumor protein p53 (TP53) (34)) and five other transcripts known to be associated with neuronal development (*Forkhead box protein A2* (FoxA2), *Cellular Retinoic Acid Binding Protein 1* (Crabp1), *Transforming growth factor beta* (TGF-β), *Catenin-beta-like 1* (Ctnnbl1) and *Ribosome Binding Protein 1* (Rrbp1) (61–63)) for further analysis. As expected, SMA MNs express markedly reduced levels of SMN compared to control MNs and DNs from control and Parkinson's disease patients (Figure 4C). Moreover, the SMA motor neurons show enhanced expression of pluripotency-related genes (OCT4, TERT, Nanog), while gene sets required for neuronal differentiation are specifically downregulated in these cell types compared to Parkinson's disease DNs. Specific downregulation of TP53, which is known to be required for efficient iPSC generation from skin fibroblasts, was also apparent in the SMA MNs (34). In agreement with this, RNA-seq analysis of total RNA extracted from mouse FACS-purified ESC-MNs demonstrates that SMA MNs show significant defects in transcripts encoding factors affecting processes critical for normal neuronal development and maintenance (61).

Interestingly, a more recent whole transcriptome analysis of human FACS-purified iPSC MNs demonstrated that SMA MNs are, in addition, chronically more hypersensitive to ER stress than healthy control MNs (62). To determine whether this phenotype is specifically related to SMA disease or a general consequence of neuronal defects, we measured changes in the levels of two selected transcripts, ATF6 and CASP3, in the different disease models by qRT-PCR analysis. ATF6 and CASP3 are both pro-apoptotic members of the unfolded protein response pathway, which are known to be specifically upregulated in MNs in response to ER stress (62,64). In agreement with this study, both transcripts are specifically enhanced in SMA MNs compared to healthy control MNs (Figure 4D). However, similar changes in ATF6 and CASP3 gene expression are also observed in Parkinson's disease patients’ DNs. These data are consistent with a recent study demonstrating a link between ER stress upregulation and impairment of protein homeostasis in Parkinson's disease DNs (40,65).

These findings support the notion that vU1/U1 levels play a critical role in neuronal development and perturbation of vU1/U1 stoichiometry contributes to the motor neuron pathophysiology of SMA patients.

## DISCUSSION

Although most of the key molecular players known to be involved in pluripotent stem cell maintenance are proteins, there is now increasing evidence that ncRNAs, in particular miRNAs and long ncRNAs, also play significant roles in influencing pluripotent stem cell behavior (66–69). Dramatic changes in splicing/polyadenylation patterns are typically associated with human ESC differentiation and primary cell reprogramming (70–72). This would suggest that additional mechanisms are key modulators of normal ESC biology. U-snRNAs are well known for their role in mRNA processing but their specific role(s) in human ESC pluripotency is largely unexplored. Expression profiling studies highlight a general trend for ESCs isolated from many different species to express numerous, often low abundance, variant forms of U-snRNAs. As development progresses, the variant U-snRNAs are down-regulated in favor of single dominant U-snRNA forms, which are maintained into adulthood (15,20,73,74). These results suggest that variant U-snRNAs may contribute to ESC maintenance and/or pluripotency decisions.

With this in mind, we analyzed the expression patterns of a newly described set of U-snRNAs, namely vU1s (25), during human ESC differentiation and somatic cell reprogramming. We have shown that vU1s are upregulated in human ESCs and become downregulated upon differentiation. In addition, these ncRNAs are upregulated following reprogramming of fibroblast into iPSCs. Taken together, these results support the idea that vU1s play specific roles in stem cell biology.

Interestingly, comparison of vU1 and U1 levels reveals specific expression patterns across different cell types. The mechanism(s) controlling these contrasting patterns of expression are currently not known but are likely to involve factors regulating vU1/U1 gene expression at multiple levels. Quantitation of nascent levels of the different U1 populations in this report suggests that U1/vU1 genes are differentially regulated at the level of transcription during differentiation and cell re-reprogramming. In agreement with this, developmental regulation of mouse vU1 genes (U1b) requires sequences located downstream of the Proximal sequence element (PSE) regulatory element within the vU1 promoter region (22). However, it is also well known that the steady state levels of U1 are vastly different from vU1 levels across the different cell types (100- to 1000-fold) despite the fact that their nascent levels are comparable in some cell types (75). These data highlight the importance of post-transcriptional processing events in regulating the relative abundance of vU1/U1 snRNA repertoires in different cell types. Consistent with this idea, a recent report describes a U1-specific Sm assembly pathway, involving U1-70k, that enhances Sm-core assembly specifically on U1 while also inhibiting assembly on other U-snRNAs (76). This pathway is believed to promote U1 overabundance and contribute to the regulation of other U-snRNA repertoires. In agreement with this idea, interference with this assembly pathway leads to dramatic reductions in U1 levels and increases in the levels of all other U-snRNAs tested. The existence of this alternate assembly pathway would explain why U1 is more susceptible to reductions in SMN levels compared to the vU1s, as some vU1 snRNPs are known not to contain the U1-70K factor (25). Furthermore, these data also raise issues regarding conclusions drawn from studies involving U1 interference assays. For example, changes in RNA processing events, following reduction in U1 levels, may have been erroneously interpreted as U1 specific when in fact they could be applicable to changes in the repertoire and abundance of additional U1 species.

The diversity of alternatively spliced mRNA isoforms and percentage of unannotated RNAs expressed is substantially greater in human ESCs and iPSCs compared to their differentiated cell types (26–28). Considering that U1 is a central component of the regulatory mechanisms controlling pre-mRNA processing, including splicing and polyadenylation, it is likely that the changes in U1 levels during differentiation, reported in this study, are important for production of the specific gene expression patterns described in these earlier reports. For example, the changes in U1 levels following differentiation of human ESCs and cell reprogramming is consistent with the concomitant increase in the progressive lengthening of pre-mRNAs during differentiation (77–79). However, our data indicate there may be an additional layer of regulation, which has gone unnoticed, involving regulation of vU1 levels. vU1s, in particular vU1.8, have previously been shown to participate in pre-mRNA processing events. Many vU1s contain base changes within their 5′ end, which is required for 5′ss recognition, it is likely that vU1s participate in pre-mRNA processing events at atypical splice junctions. In support of this, ESC and iPSCs are known to express a greater diversity of novel splice junction mRNA isoforms compared to their differentiated counterparts (28). Moreover, some vU1s reach levels of abundance in human ESCs comparable to those of the minor spliceosome snRNA, U11. Thus, individual vU1s may have the potential to profoundly affect the regulation of mRNA isoform diversity in these undifferentiated cell types (55,80).

The distinct expression patterns of vU1s and U1 during human ESC differentiation and iPSC induction is a common feature of factors known to play crucial roles in establishing and maintaining cell identity. In addition to the core pluripotent stem cell factors, OCT4, NANOG and SOX2, three master splicing regulators have also been recently identified, including members of the Muscleblind-like RNA binding factors, MBNL1/2 and SON (72,81). SON directly influences splicing of core pluripotent gene transcripts, including OCT4. In contrast, MBNL1/2 expression is specifically induced during differentiation and promotes alternative splicing of transcripts known to be specifically involved in cell lineage commitment. In particular, the regulation of FOXP1 isoform expression is considered to be the crucial step in establishing MBNL1/2 as a master regulatory of stem cell biology. Understanding how vU1s contribute to post-transcriptional splicing networks will be essential in revealing how vU1 regulation impacts cell fate decisions.

Perturbations in U-snRNA stoichiometry and repertoires is thought to be the leading cause of SMA pathogenesis in humans, reinforcing the importance of maintaining the correct balance of U-snRNA in different cell types/tissues (53,54). *SMN1* is ubiquitously expressed and though many reports have documented changes in U-snRNA/U-snRNP levels with widespread pre-mRNA splicing defects in numerous transcripts in patient cells and SMN-deficient mouse tissues, it is unclear why motor neurons are particularly sensitive (51,52,82,83). Over 80% of motor neurons, seen at postmortem analysis of SMA patients, are morphologically abnormal due primarily to failures in proper differentiation leading to impairment in dendrite and axon outgrowth formation. This would support the idea that part of the neuropathology associated with SMA might be the result of deregulation of specific factors/networks associated with the orchestration of neuronal fate decisions. Furthermore, transcriptome analysis of RNA extracted from motor neurons, isolated from spinal cords of the SMN-deficient mouse model, indicates a high proportion of aberrant splicing events (51,53). In light of what we now know regarding the role(s) of vU1s in mRNA processing events, expression patterns and contributions to cell fate decisions, it is likely that illicit expression of vU1s in the wrong cell would disrupt gene expression. Since vU1 genes are typically expressed at low levels in differentiated cells, an increase in the vU1/U1 ratio by 3- to 5-fold, as observed in iPSC-derived MNs from SMA patients (Supplementary Figure S11), could have a profound impact on the fidelity and specificity of the mRNA processing machinery. Interestingly, vU1/U1 ratios in SMA patient MNs is very similar to patterns we described for human ESCs and iPSCs, supporting the idea that vU1s play role(s) in normal neuronal development and an imbalance in their levels contributes to SMA pathology. Furthermore, since many vU1s have the potential to recognize non-canonical splice junctions, perturbation of vU1/U1 ratios, specifically in motor neurons, could result in the synthesis of aberrant RNA isoforms, with pathological consequences.

## CONCLUSION

Understanding the repertoire of vU1s and their relative profiles in different human normal and diseased cells is important to determine their contributions to cell survival and human disorders. In particular, insights into mechanism(s) that regulate the patterns of vU1 snRNA gene expression throughout human pluripotent stem cell differentiation could ultimately allow us to direct differentiation of pluripotent stem cells into specific cell lineages. Moreover, understanding the biological role(s) of individual vU1s in human cells and how their dysregulation may contribute to disease is a novel avenue of research in the field of neurodevelopmental and neurodegenerative diseases. This offers an exciting challenge that could lead to the development of novel therapies to improve the health and quality of life of patients with currently untreatable neurological disorders.

## Supplementary Material

SUPPLEMENTARY DATA
