# Supplementary material for: Variant U1 snRNAs are implicated in human pluripotent stem cell maintenance and neuromuscular disease
Source: Nucleic Acids Res. 2016 Aug 17;44(22):10960–73. doi: 10.1093/nar/gkw711 (PMC5159530; doi:10.1093/nar/gkw711)
Supplement: SUPPLEMENTARY DATA [file supp_44_22_10960__index.html]

Variant U1 snRNAs are implicated in human pluripotent stem cell maintenance and neuromuscular disease — Variant U1 snRNAs are implicated in human pluripotent stem cell maintenance and neuromuscular disease — SUPPLEMENTARY DATA 

# Variant U1 snRNAs are implicated in human pluripotent stem cell maintenance and neuromuscular disease

## SUPPLEMENTARY DATA

- SUPPLEMENTARY DATA
