# Supplementary material for: Variant U1 snRNAs are implicated in human pluripotent stem cell maintenance and neuromuscular disease
Source: Nucleic Acids Res. 2016 Aug 17;44(22):10960–73. doi: 10.1093/nar/gkw711 (PMC5159530; doi:10.1093/nar/gkw711)
Supplement: SUPPLEMENTARY DATA [file supp_gkw711_nar-01180-v-2016-File003.pdf]

SUPPLEMENTARY MATERIAL

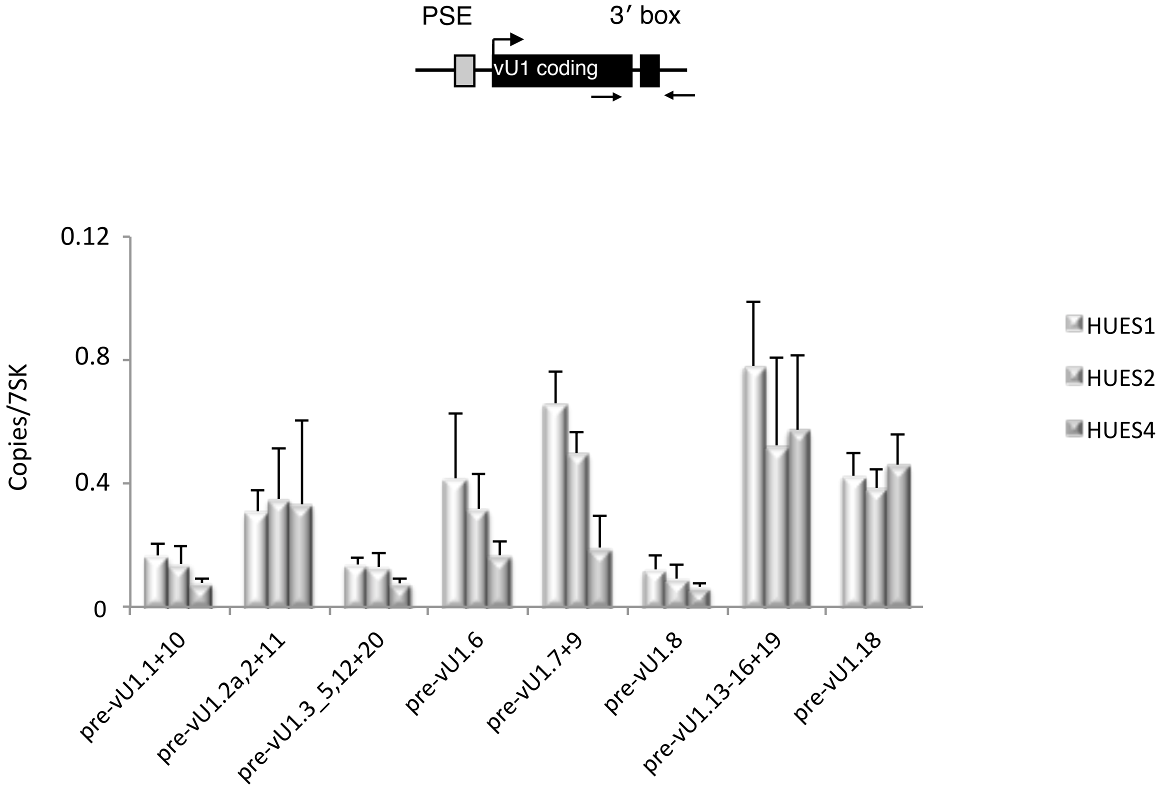

Supplementary Figure S1: Different human embryonic stem cell lines express comparable levels of vU1s

Expression profiling of nascent vU1 levels in different human ESC lines, including HUES1, HUES2 and HUES4, by qRT-PCR analysis. The position of the primers is indicated in the schematic. vU1 levels were estimated using a gDNA as standard and normalized to 7SK levels across the different cell lines. vU1 genes that show a greater than 95% sequence identity are grouped. Error bars represent standard error of 3 independent experiments.

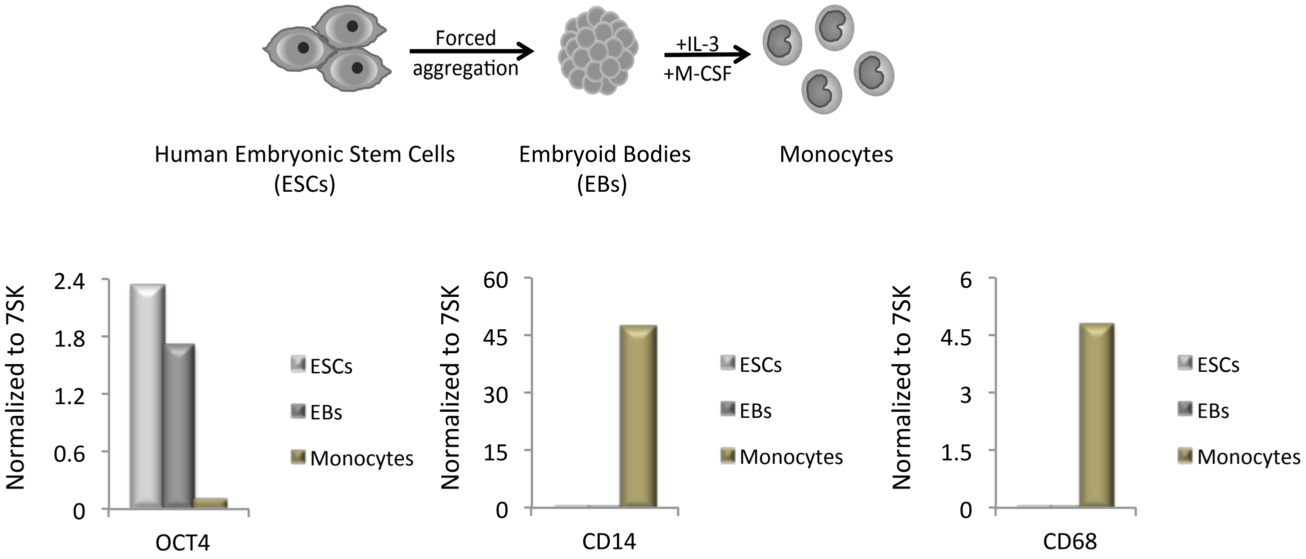

Supplementary Figure S2: Expression analysis of key pluripotent and monocytic cell markers during directed differentiation of hESCs

Schematic of the protocol used for the gradual differentiation of human ESCs (HUES2) into monocytes. Expression profiling of steady state levels of OCT4 (pluripotent cell marker) and CD14 and CD68 (monocyte/macrophage cell marker) mRNA levels in human ESCs, EBs and ESC-derived monocytes by, qRT-PCR analysis.

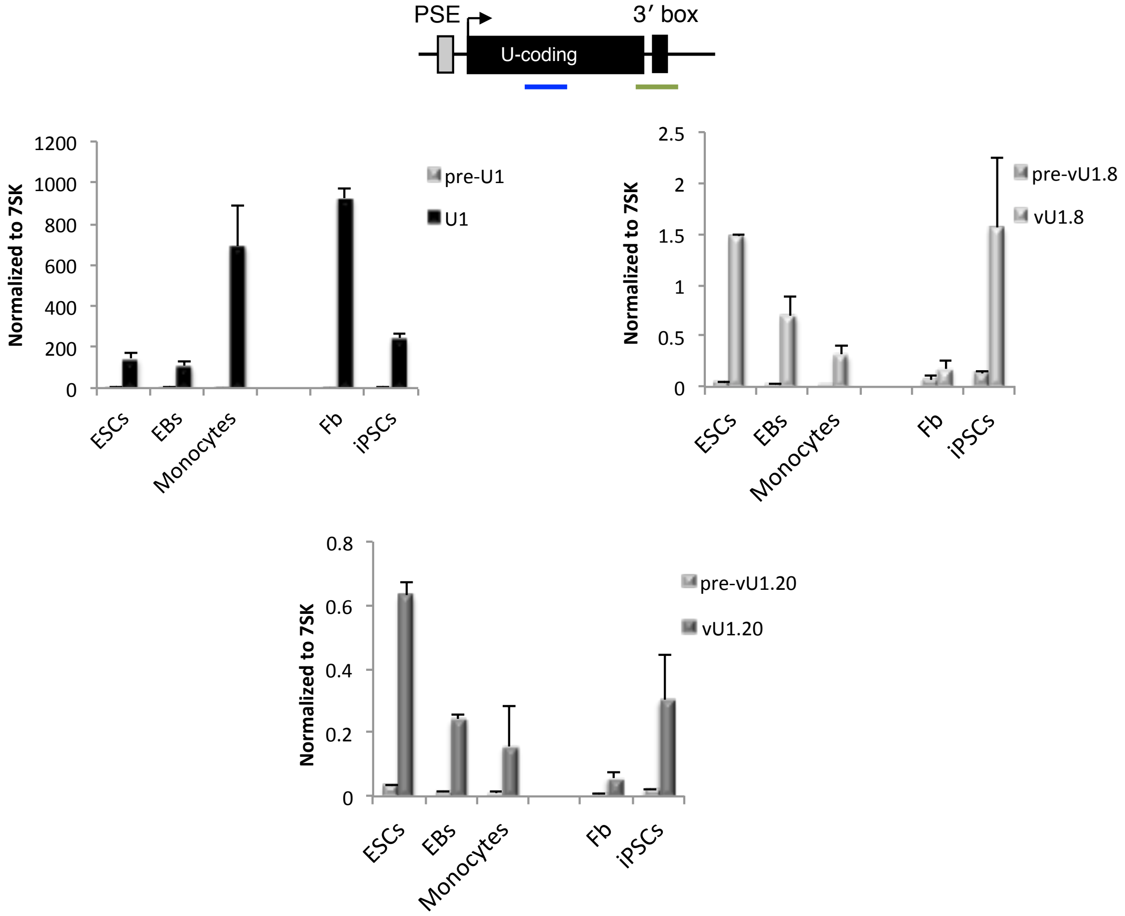

Supplementary Figure S3: U1 and vU1s are differentially expressed following differentiation and de-differentiation.

Expression profiling of immature (nascent (pre-)) and mature (processed) U1 and vU1 levels in human ESC (HUES-2), EBs, ESC-derived monocytes, human skin fibroblasts (Fb) and fibroblasts-derived iPSCs, by qRT-PCR analysis. The position of the primers is indicated in the schematic above the graphs. Primer pairs targeting the mature and/or immature transcripts are denoted as a blue and green horizontal line, respectively. vU1 levels were estimated using a gDNA as standard and normalized to 7SK levels across the different cell lines. Error bars represent standard error of 3 independent repeats.

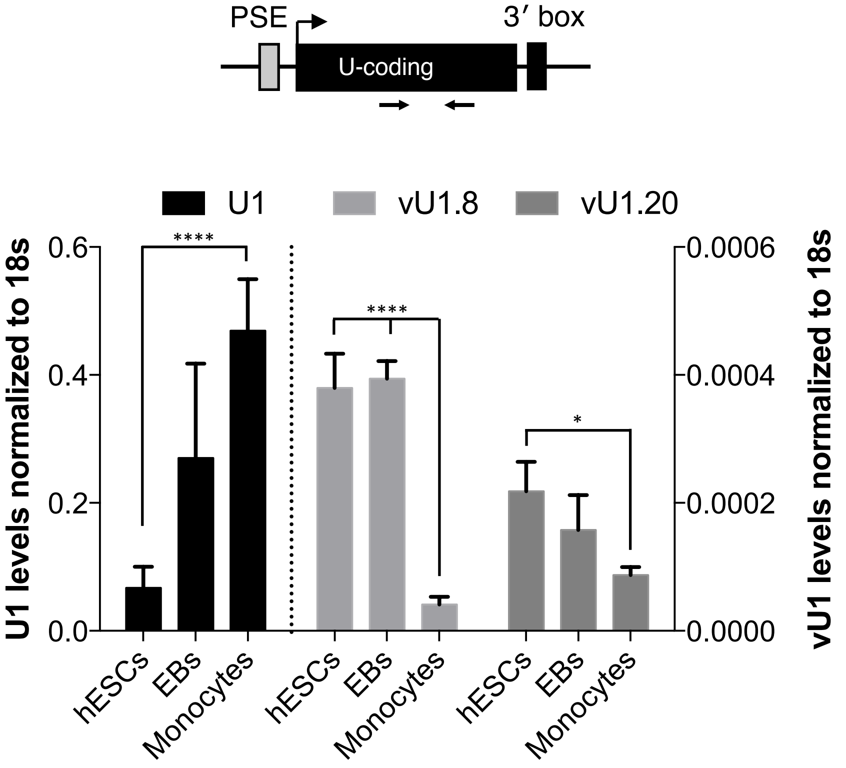

Supplementary Figure S4: vU1 expression varies inversely with U1 expression during directed differentiation of ESC into monocytes

Expression profiling of U1 and vU1 levels in human ESC (HUES-2), EBs and ESC-derived monocytes, by qRT-PCR analysis. The position of the primers is indicated in the schematic above the graphs. Levels were estimated using a gDNA as standard and normalized to the 18s ribosomal (r)RNA levels across the different cell lines. Error bars represent standard error of the mean (SEM) of three independent differentiation experiments (Two-way ANOVA analysis (U1); ****=P<0.0001 and one-way ANOVA (vU1.8 +vU1.20); ****=P<0.0001, *P= <0.05).

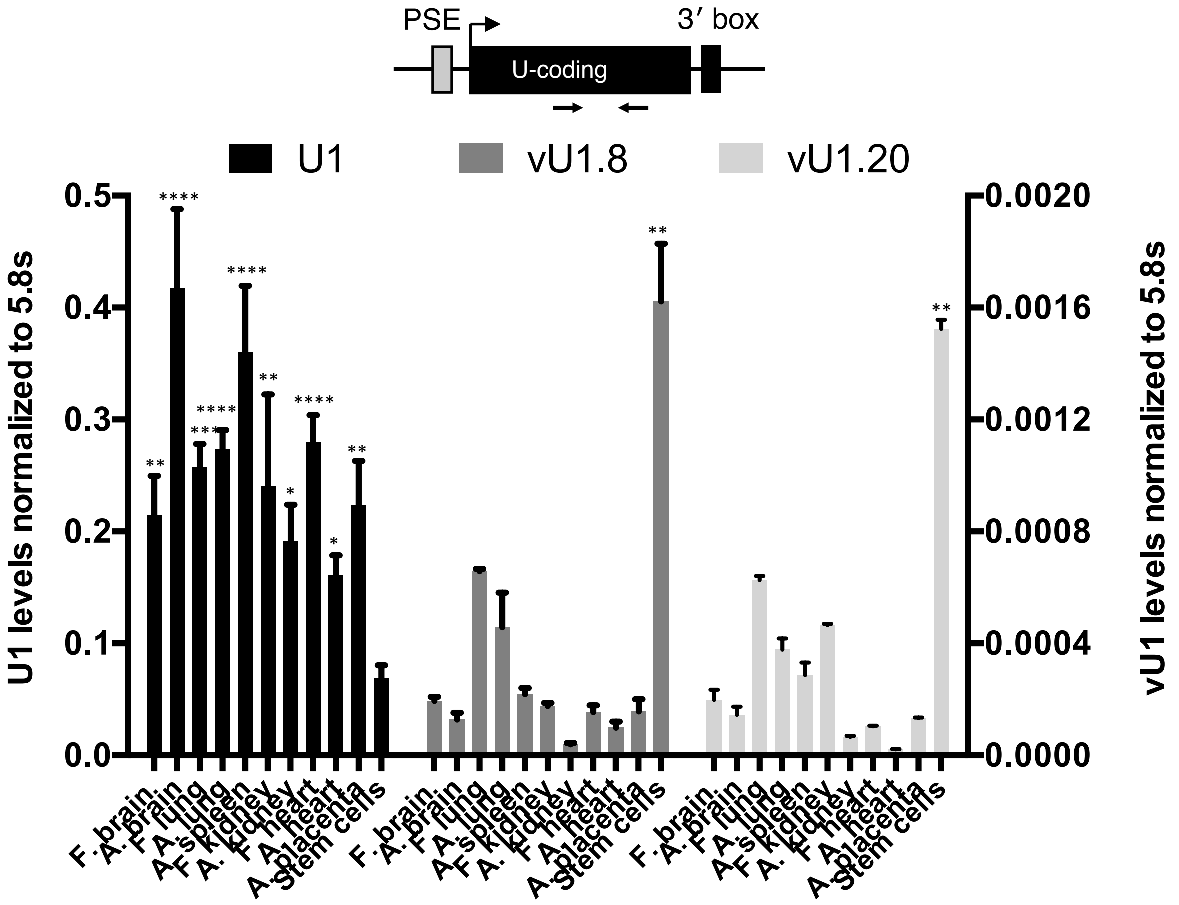

Supplementary Figure S5: Developmental switching of vU1/U1 steady state levels

Expression profiling of U1, vU1.8 and vU1.20 levels in different fetal (F) and adult tissues (A), including brain, lung, spleen, kidney, heart and placenta, by quantitative reverse-transcription (qRT)-PCR analysis. Levels of U1 are indicated on the Y-axis to the left of the graph while vU1 levels are indicated on the right Y-axis. The snRNA levels are normalized to 5.8s rRNA levels across the different cell types. The position of the primers is indicated in the schematic above the graph. Regulatory elements, known to be required for U1/vU1 expression (proximal sequence element (PSE) and 3' end processing (3'box)) are noted on the schematic. Error bars represent standard error of the mean (SEM) of 2 independent experiments (two-way ANOVA analysis; **=P>0.05, ****=P>0.0001).

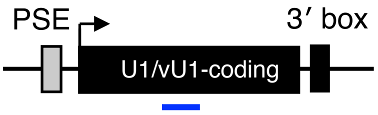

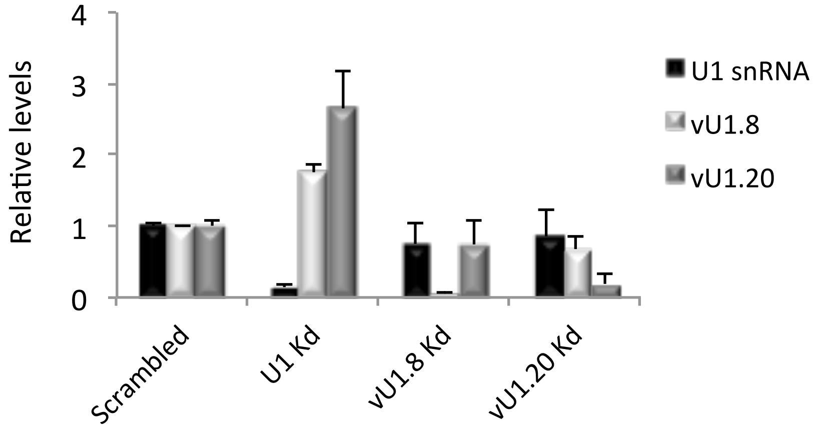

Supplementary Figure S6: U1 and vU1 levels are co-regulated

qRT-PCR analysis of U1, vU1.8 and vU1.20 levels following specific knockdown of U1, vU1.8 or vU1.20 snRNAs, with U1 or vU1 specific antisense oligonucleotides, respectively, in HeLa cells. Levels are expressed relative to levels quantitated from cells transfected with a control ‘scrambled’ oligonucleotide, which is set to 1.0. Error bars represent standard error of 3 independent repeats.

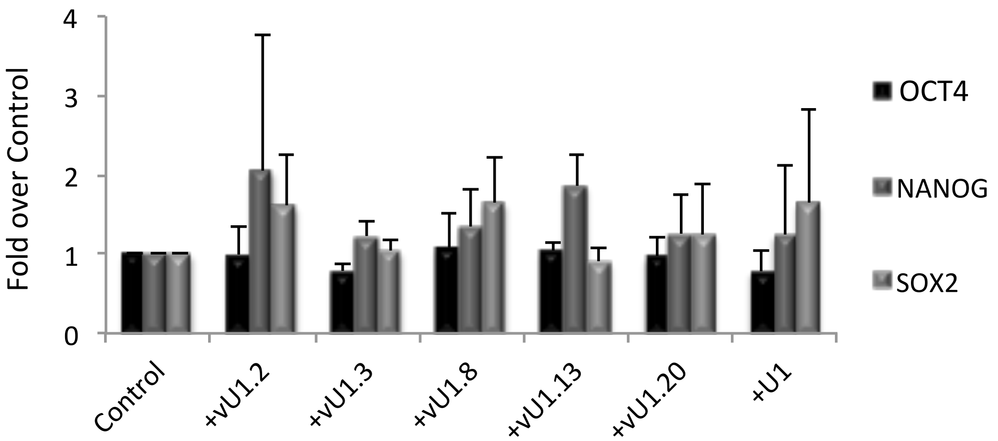

Supplementary Figure S7: Pluripotent mRNA levels are relatively unaffected following overexpression of individual vU1s in human primary fibroblasts

Changes in pluripotent cell marker mRNA levels were measured by qRT-PCR analysis, following transfection of NHDF cells with vU1 expressing plasmids, as indicated. Changes in steady state levels of OCT4, NANOG, SOX2 are expressed as fold difference over levels quantitated using a vector only control (Control). Error bars represent standard error of 3 independent experiments.

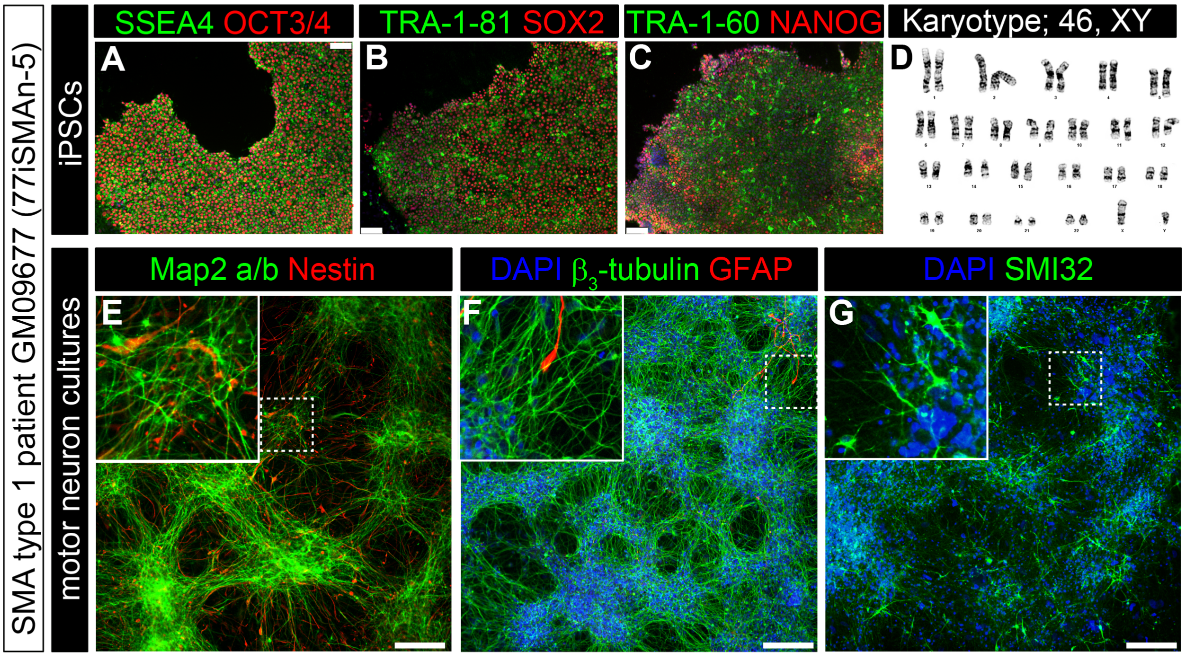

Supplementary Figure S8: Characterization of iPSC line and neuronal cultures representative of a SMA Type 1 patient iPSC line.

Positive immunostaining for pluripotency markers (**A-C**) and normal G-Band karyotype (**D**) representative of a SMA Type 1 patient iPSC line (GM09677). Upon neuronal induction the motor neuron cultures (MNs) contain few Nestin progenitors (<10%) and Map2 a/b neurons (dendritic marker) (**E**) many pan-neuronal marker β_3-tubulin (>60%) with few astrolglial (GFAP) cells (**F**), and mostly SMI32-positive motor neurons (~40%) (**G**). Scale bar for A-C is 75 μm. Scale bar for E-G 200 μm. n=5.

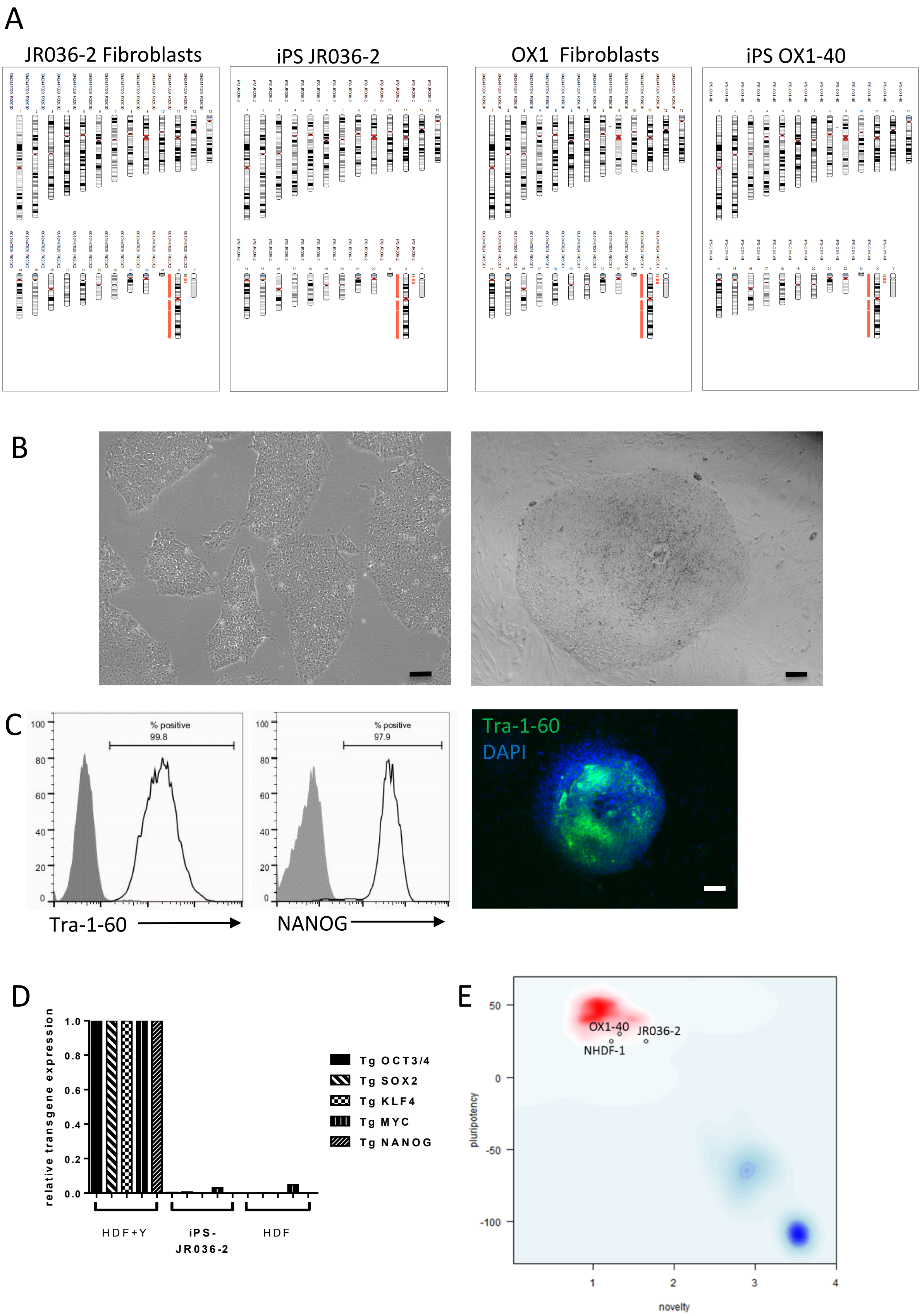

A
JR036-2 Fibroblasts
iPS JR036-2
OX1 Fibroblasts
iPS OX1-40
B
C
% positive
99.8
% positive
97.9
Tra-1-60
NANOG
Tra-1-60
DAPI
D
relative transgene expression
Tg OCT3/4
Tg SOX2
Tg KLF4
Tg MYC
Tg NANOG
HDF+Y
iPS-JR036-2
HDF
E
OX1-40
JR036-2
NHDF-1
pluripotency
novelty

Supplementary Figure S9: Characterization of JR036-2 and OX1-40 iPS cell lines. (A) SNP Karyograms of JR036-2 and OX1-40 iPSC lines (previously published parental fibroblast plots are shown for comparison), showing normal karyotype (46, XX) and absence of detectable copy number variations (single or lower copy number orange-red, higher copy number green). (B) iPSC lines show the expected iPSC colony morphology with high nucleus to cytoplasm ratio by phase-contrast microscopy, JR036-2 is on left, imaged feeder-free, OX1-40 on right, imaged on feeders, scale bar = 100 μm. (C) iPSC express expected pluripotency proteins, as shown by FACs for Tra-1-60 and Nanog for iPS JR036-2 (open black, or green plot represents antibody, filled grey or red plot represents isotype control), and by immunocytochemistry for Tra-1-60 for iPS OX1-40; (D) Retrovirus silencing in iPS JR036-2 by qRT-PCR, relative to fibroblasts infected with reprogramming vectors for 5 days, and to uninfected fibroblasts. (E) Pluritest analysis of transcriptome data shows that iPS JR036-02 and iPS OX1-40 cluster with previously published iPS NHDF-1 in the top left quadrant, consistent with a pluripotent profile.

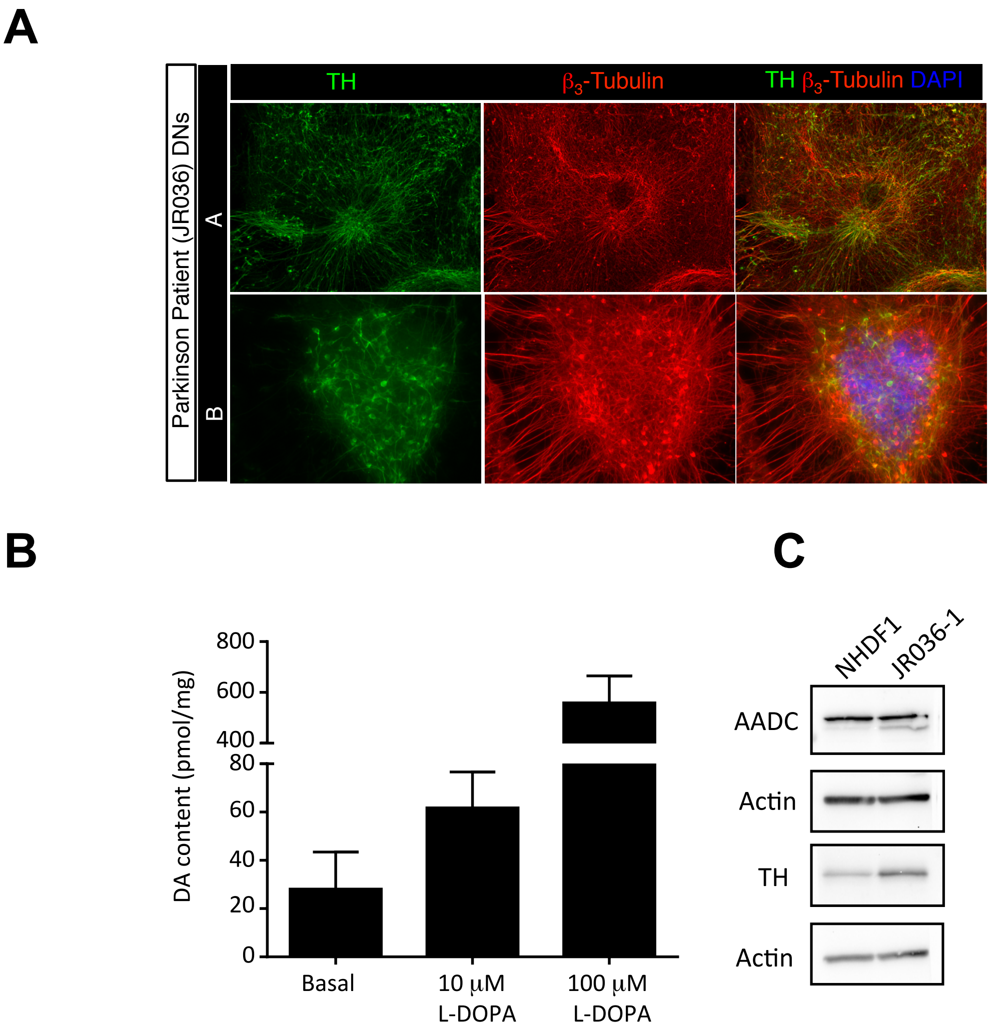

Supplementary Figure S10: Characterization of neuronal cultures derived from skin fibroblasts taken from healthy and Parkinson's disease patients.

A) Immunohistochemistry representation of differentiated dopaminergic neuronal cultures (DNs) from Parkinson's patient (JR036) showing high expression levels for the neuronal marker β_3-Tubulin (Tuji) (red), the dopaminergic neuron marker TH (green), and DAPI (blue). Scale bar for A is 75 μm and B is 200 μm **B**) Dopamine content of the differentiated DNs was measured by HPLC. DNs respond to increasing doses of L-DOPA indicating high AADC activity. Data shown are from 4 wells of 2 independent experiments and is expressed as mean ± SEM. **C**) Western blot analysis of proteins involved in dopamine synthesis and homeostasis in healthy Control (NHDF) and Parkinson patient (JR036) iPSC-derived DNs.

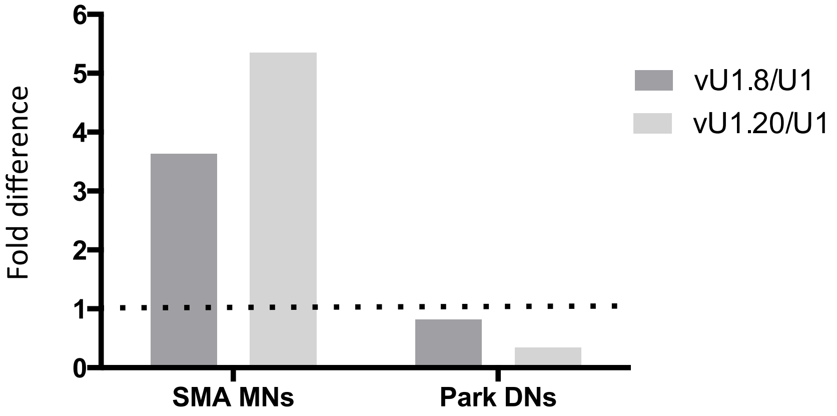

Supplementary Figure S11: Mis-regulation of vU1/U1 stoichiometry in motor neuron disease

vU1/U1 ratios are expressed as fold difference, in SMA MNs and Parkinson disease DNs, over ratios calculated in healthy control MNs and DNs, respectively, which were set to 1.0.

Supplementary Table S1: ***Cloning primers***

	**Forward**	**Reverse**
	**U1/vU1 snRNA expression cloning (pGEM4)**	
U1 Pr	5'-gagaattcgggagagaacagacgcaggggcg-3'	5'-ctggatcctgggcctctgccccgacacag-3'
U1 Cod	5'-gaggatcccatacttacctggcaggggag-3'	5'-gatctagaaagtcaggggaaagcgcgaacgcag-3'
vU1.2 Cod	5'-gaggatcccatatttacttggcaggggagataa-3'	5'-gatctagaaagtcaggggaaagcgcgaacgcag -3'
vU1.3 Cod	5′-gaggatcccatatttatctggcagaagaaatg-3′	5′-gatctagaaagtcaggggaaagcacaaactgttc-3′
vU1.8 Cod	5'-gaggatcccacatttacctagcagaagaaaaatcg-3'	5'-gatctagaaagtcgggggaaaagagcgaacgcag-3'
vU1.13 Cod	5′-gaggatcccatacttacctggcaggggag -3′	5′-gatctagaaagtcagaggaaagcgcgaacgcag-3′
vU1.20 Cod	5′-gaggatcccatgtttatctggcagaagaaatg-3′	5′-gatctagaaagtcaggggaaaacacagacacagttc-3′
U1 3′end	5'-ctagagtttctaaaagtagactgtacgctaag	5'-agcttcaaccaagacacaaaccaaaacaaaaaa
	ggtcatatctttttttgttttggtttgtgtcttggttga-3'	agatatgacccttagcgtacagtctacttttagaact-3'

Supplementary Table S2: *qPCR primers.*

Name	Forward	Reverse
	**Nascent vU1 snRNA oligonucleotides**	
vU1.1+10	5'-gaaactcgactgcataatttgtggtag-3'	5'-cacatacaatctgcgttttccagcac-3'
vU1.2a, 2+11	5'-gaaactcgactgcataatttgtggtag-3'	5'-ggagcgtacggtctatttttgaaacc-3'
vU1.3-5,12+20	5'-gaatctcgactacataatttgtggcag-3'	5'-cagcaaacaaaatccacttttaaaacgac-3'
vU1.6	5'-gaaacttgactgtataatttgtggcagtg-3'	5'-gcagcaaacaaaacctatttttaaaacgac-3'
vU1.7+9	5'-gaaactcgactgcataatttgtggtag-3'	5'-ccttataggggagtctattttttcattac-3'
vU1.8	5'-gaagctaattcgtgcaacttccc -3'	5'-gagcgaacgcagtctc -3'
vU1.13-16+19	5'-ggaactcgactgcataacttgtgatagt-3'	5'-cggctcacctactattcgcaccac-3'
vU1.17	5'-gaaactcgactgcataatttgtggtag-3'	5'-ctttgcataccaactgctttttgcactg-3'
vU1.18	5'-gaaactcgactgcataatttgtggtag-3'	5'-cccttgctgtgtagactattctttaaac -3'
	**snRNA/mRNA oligonucleotides**	
U1	5'-caggggagataccatgatcacgaag-3'	5'-ggtcagcacatccggagtgcaatgg-3'
vU1.8	5'-cagaagaaaaatcgtgtttac-3'	5'-gaagttgcacgaattagc-3'
vU1.3	5'-tctggcagaagaaatgttatgatc -3'	5'- gggaaagcacaaacagttcc-3'
vU1.20	5'-gtttatctggcagaagaaatgttatg-3'	5'-gggaaaacacagacacagttc -3'
hCD14	5'-atcgaccatggagcgcgcgt-3'	5'-aaggcttcggaccagtcggg-3'
hCD68	5'-gtcatggaaatgccacggttc-3'	5'-aggttgggctaggactcgg-3'
hOCT4	5'- cgagcaatttgccaagctcctgaa -3'	5'-ttcgggcactgcaggaacaaattc -3'
hNANOG	5'-cctatgcctgtgatttgtgg -3'	5'-aagtgggttgtttgcctttg -3'
hSOX2	5'-acaccaatcccatccacact -3'	5'-cctccccaggtttttctctgt -3'
hTERT	5'-cctgctcaagctgactcgacaccgtg-3'	5'- ggaaaagctggccctggggtggag c-3'
hCRABP1	5'-gtgaaggcagctggcttgc-3'	5'-cgggatcacctaatattggtag-3'
CTNNBL1	5'-gcagagaacatcggggacg-3'	5'-gagagagtccatgatgcgcag 3'
RRBP1	5'-caagatggcagccttcgaagtg 3'	5'-cgttgctcctgtcgccacag-3'
hTGFβ	5'-cacatataccagtggtgatcag-3'	5'-gcacgcttcttccgccggttgg-3'

hSMN-FL	5'-cctcccatatgtccagattctctga-3'	5'-tctttttgattttgctgaaacccata-3'
hTP53	5'-ccatggcgcggacgcggg-3'	5'-ctcccctgccctcaacaagatg-3'
hFOX-A2	5'-gtgaagatggaagggcacga-3'	5'-catgttgctcacggaggagt-3'
hATF6	5'-cttttagcccgggactcttt-3'	5'- tcagcaaagagagcagaatcc -3'
hCASP3	5'-tggaattgatgcgtgatgtt-3'	5'- tggctcagaagcacacaaac-3'
	**Normalization control oligonucleotides**	
7SK	5'-ctgatctggctggctaggcggg-3'	5'-gaagaccggtcctcctctatcgg-3'
5.8s	5'-caagcgacgctcagacagg -3'	5'- gtggatcactcggctcgtgc-3'
18s	5'-gataacgaacgagactctggcatg-3'	5'-gcatcacagacctgttatt-3'

Supplementary Table S3*: 2’O Methyl Phosphorothionate Oligonucleotides.*

	**Nuclear snRNA Knockdown (2’O-Me)**
Control	5'-gaaggaactagcgtacgacggu-3'
U1	5'-gguaucteccctgccaggtaaguau-3'
vU1.8	5'-guaaacacgatttttcttcugcu-3'
vU1.20	5'-aacauttcttctgccagauaaac-3'

***Supplemental Table S4**: Primary antibodies used for immunocytochemistry, FACS and western blot analysis*

Antigen	Dilution	Catalog #	Isotype	Manufacturer
SSEA4	1:250	MAB4304	mIgG3	Millipore
TRA-1-60	1:250	09-0010	mIgM,κ	Stemgent
TRA-1-81	1:250	09-0011	mIgM,k	Stemgent
Nestin	1:1000	AB5922	Rabbit IgG	Millipore
GFAP	1:1000	Z0334	Rabbit IgG	DAKO
OCT4	1:250	09-0023	Rabbit IgG	Stemgent
NANOG	1:250	09-0020	Rabbit IgG	Stemgent
SOX2	1:500	AB5603	Rabbit IgG	Millipore
TuJ1 (β_3-tubulin)	1:1000	T8535	mIgG2b	Sigma
SMI32	1:1000	SMI-32R	mIgG1	Covance
Map2 a/b	1:550	M 1406	Mouse IgG	SIGMA
βIII-Tubulin	1:500	MMS-435P	Mouse 1gG	Covance
TH	1:500	AB152	Rabbit IgG	Millipore
IgG (Alexa Fluor® 647 Conjugate)	1:15	2985S	Rabbit IgG	Cell Signaling
NANOG (Alexa Fluor® 647 Conjugate)	1:15	5448S	Rabbit IgG	Cell Signaling
AADC	1:1000	ab1569	Rabbit IgG2b	Millipore
TH	1:500	ab1542	Sheep IgG1	Millipore
β-actin	1:0000	ab8227	Rabbit IgG	Abcam
